# Systemic approaches for the protection of our oceans and marine environments

**DOI:** 10.1038/s44168-026-00341-x

**Published:** 2026-01-28

**Authors:** Phoebe Koundouri, Ebun Akinsete, Tony Capon, Rita R. Colwell, Adel S. El-Beltagy, Ismahane Elouafi, Jim Falk, Alberto Naveira Garabato, Charles F. Kennel, Conrad Landis, Margaret Leinen, Ali Mashayek, Cherry A. Murray, Ismail Serageldin, Kazuhiko Takeuchi, Makoto Taniguchi, Eleni Toli

**Affiliations:** 1 Athens University of Economics and Business, Athens, Greece; 2 University of Cambridge, Cambridge, UK; 3 ATHENA Research and Innovation Centre, Athens, Greece; 4 Monash Sustainable Development Institute, Monash University, Melbourne, VIC Australia; 5 School of Public Health and Preventative Medicine, Monash University, Melbourne, VIC Australia; 6 Center for Bioinformatics and Computational Biology, University of Maryland, College Park, MD USA; 7 Johns Hopkins Bloomberg School of Public Health, Baltimore, MD USA; 8 International Dryland Development Commission (IDDC), Cairo, Egypt; 9 Arid Land Agricultural Graduate Studies & Research Institute (ALARI), Ain Shams University, Cairo, Egypt; 10 Consultative Group on International Agricultural Research (CGIAR), Montpellier, France; 11 School of Geography, Earth and Atmospheric Sciences, University of Melbourne, Melbourne, VIC Australia; 12 University of Wollongong, Wollongong, NSW Australia; 13 University of Southampton, Southampton, UK; 14 Scripps Institution of Oceanography, University of California, San Diego (UCSD), San Diego, CA USA; 15 University of Arizona, Tucson, AZ USA; 16 Harvard University, Cambridge, MA USA; 17 The Library of Alexandria, Alexandria, Egypt; 18 Institute for Global Environmental Strategies (IGES), Kanagawa, Japan; 19 Institute for Future Initiatives (IFI), The University of Tokyo, Tokyo, Japan; 20 Research Institute for Humanity and Nature (RIHN), Kyoto, Japan; 21 University of Athens, Athens, Greece

**Keywords:** Climate sciences, Ecology, Ecology, Environmental sciences, Ocean sciences

## Abstract

Protecting our oceans and advancing a sustainable blue economy require in-depth understanding of marine systems, driven by robust ocean observation, monitoring and valuation. Yet collecting reliable data remains time- and resource-intensive. This data is vital for scientists, emergency responders, and decision-makers to support early-warning systems and emerging tools like digital twins. Stronger support is therefore needed for data collection and its integration into systemic, innovative, and stakeholder-engaged ocean observation efforts.

The effective protection and sustainable use of the oceans demands the urgent integration of ocean earth science, with socio-economic science, into marine policy frameworks. As underscored by the RACC Symposium 2024 at the STS Forum in Kyoto, Japan^1^, the development of a Blue Economy framework is essential, not only to drive climate and industrial interventions but also to anchor them in sustainability through rigorous cost-benefit analysis (CBA). These can be underpinned by detailed geospatial monitoring and observation, with climatic, environmental and socio-economic modelling supported by new data-driven tools such as machine learning and digital twins.

## Methods

This brief communication was developed by adopting an interdisciplinary desktop study, focused on a literature review of academic journals, policy outputs and publicly available research project deliverables. The desk study utilised a thematic review, which ran a search of academic literature related to oceans, oceans monitoring, ocean protection, marine ecosystem protection, ocean monitoring technology, digital twins, digital twins of the ocean and marine policy. The review focused particularly on the connections between ocean protection, digital twin technologies, policy development and policy implementation. While the study reviews the most relevant literature, policy developments in the context of a rapidly changing geopolitical landscape raises the risk of publication lag.

### Data Sources

The review utilised both academic and grey literature in the form of journal articles, policy outputs, reports from governmental and multilateral agencies, obtained from multiple sources. Academic journal articles were sourced from databases, such as Scopus, Web of Science, ScienceDirect, and Google Scholar, while grey literature from international and governmental agencies such United Nations, OECD, UNEP, the European Commission, and NOAA. Several rounds of internal review for both quality and content were conducted, with revisions made based on feedback provided by co-authors.

## Ocean Observations: approaching a complex ecosystem through a “data lens”

It is widely understood that the ocean plays a vital role in regulating the climate and mitigating the consequences of climate change. In addition to being an ecological asset, the ocean is a vast and critical economic asset. Yet, until recently, its dynamic systems have remained under-monitored and undervalued, limiting both conservation and investment efforts. Data science, including data collection, analysis, sharing and reuse, and data-driven tools, contribute to advancing understanding of the value of marine and coastal ecosystems.

Existing modern technologies, such as remote sensing (of various biological, chemical and physical properties of the oceans), in-situ observations (e.g. global floats and drifter programs, mooring arrays, coastal sea level gauges, ship-based observations, and increasingly autonomous gliders) take decades to plan and develop. Furthermore, existing ocean-monitoring technologies require years, if not decades, to build the extensive records needed to support monetisation of the data and to provide the relevant services.

Such global programs, even after being deployed, whilst having demonstrated their usefulness and proved to be lucrative to industry and essential to society at large (e.g. providing extreme event predictions, hazard systems, fisheries monitoring), yet often lack stable and reliable funding. Their economic service and value are rarely captured and adequately monetised. This leaves key capacities they offer vulnerable to cuts. As a case in point, there is uncertainty around US funding, which accounts for a considerable amount of funding for the global observing system. This includes the uncertainty over funding for the National Oceanic and Atmospheric Administration’s U.S. Integrated Ocean Observing System (IOOS, a major contributor to global ocean-observing infrastructure. This could have far reaching impacts on the effective operation of emergency warning systems which rely on such observations to predict extreme weather events, such as hurricanes, tsunamis, ocean surges and other coastal hazards^2^. This integrated international ocean observation data is used by all nations not just in the case of extreme events, but also to monitor sea level rise and related changes in the coastal zone, harmful algal blooms (and their effects on aquaculture as well as human health, fisheries), pollution and biodiversity loss. The Global Ocean Observing System (GOOS) framework emphasises that consistent, sustained observing networks are essential for climate, ecosystem and economic applications^2^.

Emerging technologies, such as machine learning, new state-of-the-art networks of sensors in the ocean interior, and Digital Twins of the Ocean (DTOs), are revolutionising our ability to model, monitor, and monetise changes in marine environments. We argue that:

I. Our existing ocean monitoring technologies are drastically undervalued. While these technologies underpin core activities related to climate change adaptation, their availability is often limited by lack of funding, integrated data systems, and public awareness. Better appreciation (valuation) of existing technology and capitalising on the emergent ones can help transform technology implementation and create much-needed infrastructure for sustainable ocean economy strategies;
II. The time to act is now. Our oceans and the marine environments are facing accelerating threats (biodiversity loss, warming, acidification, extreme events, such as sea level rise and heatwaves), while the recent perturbations to trans-Atlantic geopolitics, expose how fragile global observation and hazard prediction systems are. These trends compound risk across food security, coastal infrastructure, human health, and economic sectors reliant on marine goods and services, and are forcing nations to rethink continental and international economic, security, and research frameworks.

In RACC 2024, DTOs were discussed extensively in the context of the above revolution. We discuss them further below.

## What Are Digital Twins of the Ocean?

Digital Twins are dynamic virtual representations that integrate real-time data, predictive modelling and visualisation to replicate and simulate physical systems in order to support the observation of ocean systems and the deployment of suitable solutions. When applied to marine environments, DTOs integrate satellite data, sensor outputs, modelling, and human knowledge to simulate the ocean’s physical, chemical, biological, and economic processes. They are a transformative technology that empowers the replication of ocean conditions with unprecedented granularity. They allow all stakeholders, from governments and policymakers to researchers, industries and investors, to pose “what if” questions and test scenarios, predict impacts of policy, economic activities or climate changes, and optimise resource management.

Key features of DTOs include the integration of real, multi-dimensional data and models, with continuous data flow, and scenario simulation. Unlike traditional ocean monitoring, DTOs emphasise interactivity, stakeholder engagement and co-design with industry from the outset, ensuring that tools address real-world needs. Whilst this brings about new potentials for progress, it poses challenges in acquiring sufficient data, ensuring sharing of data under the FAIR principles, addressing computational power demands, whilst protecting research freedom and aligning to governance and policy frameworks.

The European Commission has prioritised DTOs under its *Digital Twin of the Ocean* initiative^3^, particularly under the “Mission Restore Our Ocean and Waters by 2030, building a critical link between science and society. The EU is building infrastructure (e.g. the recently completed EDITO-Infra and the EDITO-Model Lab projects) that enables scenario modelling, user-driven applications, and public access to ocean data.

## Yes, but is there supporting evidence that DTOs could help?

EU efforts have already delivered first results. Real-world marine DTO pilots exist and are delivering measurable monitoring outcomes. In the EU-funded ILIAD initiative, a pilot called “Water Quality DTO” in the Trondheim Fjord (Norway) uses real-time surface and benthic sensor data, autonomous underwater vehicle inputs, and numerical models to detect micro-plastics and monitor algae blooms^4^. This shows that DTOs can move beyond theoretical constructs into operational environmental monitoring, and from there to valuation and investments.

Taking a closer look at the manufacturing sector, which is the most advanced in adopting Digital Twin technologies, we can prove that Digital Twins can move beyond experimental prototypes into full operational use. Manufacturers now use virtual factories to simulate entire production lines, perform rapid “what-if” tests, anticipate maintenance needs, and optimise entire supply chains in real time. This practical maturity offers a useful blueprint: if an industry as complex as manufacturing can embed twin-technology into core operations, then with proper investment and infrastructure, similar DT approaches, such as for our oceans, can also transition from proof-of-concept to decision-ready platforms.

## Fostering sustainable blue economy through monitoring and valuation

The value of DTOs goes far beyond research purposes. The availability of continuous, high-resolution data allows us to estimate the economic consequences of various phenomena and activities related to the ocean, whether it is the impact of degradation or the benefits of restoration, fisheries productivity, offshore energy production, maritime industry, coastal or marine tourism, and many more. Accurate and continuous monitoring has become, in all these cases, an essential economic asset, providing the basis for valuation of ecosystem services and allowing for data-driven assessments and accurate forecasts.

## Integrating Economic Valuation and Digital Innovation in Marine Governance

The integration of economic reasoning into marine governance is increasingly enabled by the convergence of CBA, ecosystem service valuation, and digital tools, such as DTOs. These approaches provide a framework for assessing the ecological and economic trade-offs inherent in marine and coastal policy decisions. Recent advances show that coupling spatial data with economic valuation methods can transform fragmented management practices into more coherent and evidence-based ocean strategies.

## Mapping Ecosystem Services for Informed Decision-Making

A foundational requirement for effective marine valuation^5–7^ is the accurate and spatially explicit mapping of underlying ecosystem services. Spatial models that connect biophysical data (for example, habitat distribution, hydrological cycles, and biological productivity) with valuation techniques (such as benefit transfer methods, discrete choice experiments, and contingent valuation) allow for the monetisation of services, such as coastal protection, nutrient cycling, carbon storage, recreational opportunities, and biodiversity maintenance.

These spatially grounded tools enable researchers and policymakers to quantify not only the flow of ecosystem services but also their distribution across geographic areas and social groups. This is essential for understanding who benefits from ocean resources and who bears the cost of degradation, thereby informing more equitable and sustainable management strategies.

## Enhancing Predictive Capacity Through Digital Twins of the Ocean

DTOs represent a transformative technology that brings together real-time monitoring, predictive modelling, visualisations and scenario analysis^8^. When ecosystem service valuation models are linked to this transformative technology and the dynamic platforms of the DTOs, it becomes possible to simulate policy impacts with unprecedented resolution. Policymakers can test alternative governance strategies, evaluate economic implications under different climate scenarios, and optimise interventions, such as offshore wind deployment, aquaculture planning, or conservation zoning.

This integration is particularly powerful in enabling “what if” analyses and long-term forecasting that factor in both ecological and economic outcomes. It also supports the development of marine investment ecosystems that are responsive to shifting environmental baselines and policy objectives.

## Indicators and Visualisation for Marine Policy Assessment

Key Performance Indicators (KPIs) that capture ecological, economic, and social dimensions of marine systems, are essential tools for policy assessment. Increasingly, these indicators are derived from geospatial datasets collected through satellites, in situ observations, and ecological models. These datasets serve as the basis for evaluating the cumulative and long-term effects of marine activities.

GIS-based ecosystem service models, in particular, provide a powerful means of visualising trade-offs and synergies across sectors, such as tourism, fisheries, energy, and shipping. These visualisations aid in transparent and participatory decision-making, enabling stakeholders to co-design and co-evaluate marine spatial plans and climate adaptation strategies to support a sustainable Blue Transition

## Engaging Stakeholders in the Blue Transition

With the impacts of human activity being the primary cause of ocean degradation, people are central to the solution. Actively and effectively engaging blue economy stakeholders through participatory frameworks that blend both interdisciplinary and transdisciplinary methods is essential^9^. Local and expert knowledge can be leveraged not only to define key challenges faced, but also to co-design innovative solutions and strategies to protect marine ecosystems while developing a sustainable blue economy^10^. Adopting systemic frameworks, such as the Systems Innovation Approach and Transition Management allows for the engagement of stakeholders from various sectors while utilising tools, such as DTOs to develop a holistic understanding of the issues and facilitate co-creation of transition pathways towards a sustainable blue economy. In this way an enabling environment can be created to not only actively support the implementation of policies geared towards the protection of marine ecosystems but also drive blue entrepreneurship.

## What should policy-makers and the research community prioritise?

Investment in long-term ocean observing systems is crucial. These systems, operating from local to global scales, provide essential data for developing effective climate mitigation and adaptation strategies. The use of this data can be maximised via new technologies, such as Digital Twins for Oceans which are more than scientific tools; they are investment enablers, offering decision support through effective ocean observation. By coupling monitoring with economic valuation, DTOs unlock new pathways for sustainable blue finance.

In light of the main outcomes of the third United Nations Ocean Conference (UNOC3, 2025) it is clear that the opportunities unlocked by innovative use of data for ocean observation are essential. However, this must be coupled with both the data infrastructure and governance models to realise their full potential^11^. It is therefore important to further support the DTO development, as a core part of EU and national ocean strategies, aligning with the Digital Ocean initiatives under the European Green Deal.

As data lies at the very core of the DTOs, incentives for data sharing should be created. In this respect, the building of open, standardised and interoperable infrastructures that support integration and reuse of results is equally important, as these infrastructures can accelerate digital twin development and use, and increase the value of shared data and services.

In all the above, inclusivity and access are central as the ocean belongs to all nations. It is important to promote international cooperation (particularly with nations in the global south and at risk indigenous communities in Small Island Developing States) to support the development of frameworks that expand the collection and harmonisation of data for ocean observation, while fostering local marine stewardship through stakeholder engagement and citizen science^11^. Stakeholder engagement as part of the process of DTO development and deployment is important. Only by applying methodologies that prioritise stakeholder co-design^12,13^ can we ensure that digital twins reflect stakeholders’ needs and priorities and the realities of the local ecosystems.

## Data Availability

No datasets were generated or analysed during the current study.
